# Differential Influence of Clonal Integration on Morphological and Growth Responses to Light in Two Invasive Herbs

**DOI:** 10.1371/journal.pone.0035873

**Published:** 2012-04-27

**Authors:** Cheng-Yuan Xu, Shon S. Schooler, Rieks D. Van Klinken

**Affiliations:** CSIRO Ecosystem Sciences, Dutton Park, Brisbane, Queensland, Australia; Jyväskylä University, Finland

## Abstract

**Background and aims:**

In contrast to seeds, high sensitivity of vegetative fragments to unfavourable environments may limit the expansion of clonal invasive plants. However, clonal integration promotes the establishment of propagules in less suitable habitats and may facilitate the expansion of clonal invaders into intact native communities. Here, we examine the influence of clonal integration on the morphology and growth of ramets in two invasive plants, *Alternanthera philoxeroides* and *Phyla canescens*, under varying light conditions.

**Methods:**

In a greenhouse experiment, branches, connected ramets and severed ramets of the same mother plant were exposed under full sun and 85% shade and their morphological and growth responses were assessed.

**Key results:**

The influence of clonal integration on the light reaction norm (connection×light interaction) of daughter ramets was species-specific. For *A. philoxeroides*, clonal integration evened out the light response (total biomass, leaf mass per area, and stem number, diameter and length) displayed in severed ramets, but these connection×light interactions were largely absent for *P. canescens*. Nevertheless, for both species, clonal integration overwhelmed light effect in promoting the growth of juvenile ramets during early development. Also, vertical growth, as an apparent shade acclimation response, was more prevalent in severed ramets than in connected ramets. Finally, unrooted branches displayed smaller organ size and slower growth than connected ramets, but the pattern of light reaction was similar, suggesting mother plants invest in daughter ramets prior to their own branches.

**Conclusions:**

Clonal integration modifies light reaction norms of morphological and growth traits in a species-specific manner for *A. philoxeroides* and *P. canescens*, but it improves the establishment of juvenile ramets of both species in light-limiting environments by promoting their growth during early development. This factor may be partially responsible for their ability to successfully colonize native plant communities.

## Introduction

Plant invasion is a threat to biotic diversity and ecosystem services worldwide [1], [2], [3]. Many invasive plants can readily propagate clonally, and this can contribute to their invasiveness [4], [5]. Resource sharing among inter connected ramets, i.e. clonal integration [6], [7], [8], [9], [10], [11], [12], [13], buffer the spatial variation in environmental conditions [14], and may increase the performance of clonal plants over co-occuring species that are non-clonal or with little integration [15], [16]. For example, vegetative propagation with ramets that are connected to, and can exchange resources with, a well-established introduced mother plant may help facilitate colonization, particularly in habitats presently occupied by an intact native community. Although whether clonal integration increases the competitive ability of plant invaders remains controversial [16], [17], [18], [19], [20], [21], [22], there is clear evidence that it improves the establishment (including survival and early stage growth) of juvenile ramets of plant invaders in habitats with low resource availability, adverse environmental conditions, or intense competition [20], [23], [24], [25], [26], [27]. These studies mostly contrasted the performance of connected and severed ramets (e.g. survival rate, biomass, stem number), but rarely examined the effect of clonal integration on morphological traits that are closely associated with acclimation to adverse conditions. In addition, the time effect of clonal integration, i.e. whether the influence of clonal integration on daughter ramets decrease as the daughter ramets establish, is rarely studied [28].

Spatial environmental heterogeneity is very common in natural habitats. Reciprocal translocation among ramets exposed to different resource conditions can buffer spatial variation in resource availability and facilitate efficient acquisition of heterogeneously distributed resources [14]. The positive influence of clonal integration on the establishment of juvenile ramets in heterogeneous habitat has primarily been observed under field conditions. However, experimental tests under controlled conditions remain limited [29]. Size disparity between the mother plant and daughter ramets is also common in field conditions. For example, a mother plant established on a patch with high resource levels may be many times larger than the daughter ramets expanding into neighbouring habitat. Presumably, mother plants are better able to acquire resources due to their larger size and their dominance of the patch they have occupied. They also have larger resource demands, e.g. investment in their own branch growth, than the connected daughter ramets. Questions such as how the mother plant allocates resources between investment in branch and ramet growth are rarely addressed in the literature.

In this study, we investigated two invasive clonal plants, *Alternanthera philoxeroides* (Martius) Grisebach (common name: Alligator weed, Amaranthaceae) and *Phyla canescens* (Kunth) Greene (common name: lippia, Verbenaceae). These two species are perennial herbs native to South America and invade wetland habitats in Australia. They have successfully invaded, and become the dominant species in some communities that originally included many non-clonal plant species [30], [31]. Both species are typically more prostrate than co-occurring grasses and forbs (Xu, personal observation), seemingly making juvenile ramets vulnerable to intensive competition for light. For example, *P. canescens* was introduced as a “mowless” lawn for its low growth habit [32] and seedling density and survival is greatly reduced in the presence of existing vegetation under natural conditions [33]. Although there is evidence that these invaders can be shade tolerant, the growth of severed, shaded ramets are still significantly reduced [34]. Thus, subsidy of assimilates from a mother plant in adjacent unoccupied spaces may play an important role in supporting the recruitment of daughter ramets in occupied habitats, thereby facilitating their competitive dominance over native species.

We addressed the effect of clonal integration on ramet physiology in *A. philoxeroides* and *P. canescens* in a previous study and found a species-specific pattern in the amount of maternal carbohydrate subsidy under contrasting light levels [35]. However, morphological traits, which are important to ramet light acclimation, were not investigated. Here, we designed a greenhouse experiment to simulate the expansion of daughter ramets and unrooted mother branches from one mother plant established in the absence of competition into neighbouring habitats with contrasting light conditions. This was a companion experiment to the previous study conducted on different plants [35]. We compared reaction norms of morphological and growth traits to contrasting light levels of severed ramets, connected ramets and branches from the same mother plant of *A. philoxeroides* and *P. canescens*. This allowed us to assess the effect of clonal integration on light responses of both ramets (including through time) and the way mother plants prioritised resource investment between branches and ramets. In each species, we tested five hypotheses: (1) connected ramets grow faster than severed ramets; (2) the positive effect of clonal integration on the growth of daughter ramets is greater in shade than in full sun; (3) early growth of daughter ramets is mainly affected by clonal integration, not light level; (4) connected ramets display less morphological plasticity to contrasting light conditions (indicated by the light reaction norm) than severed ramets; and (5) under the same light condition, connected ramets display greater growth than branches of mother plants. Because clonal plants often display labor division between ramets to capture locally abundant resources (see review [14], [36]), mother plants might allocate more resources to subsidize (a) the growth of full-sun ramets, irrespective of whether they are rooted, to promote light harvest, or (b) the growth of daughter ramets (rather than non-rooted branches) to facilitate underground resource assimilation. The first prediction can be an alternative hypothesis to our null hypothesis 2 while the second prediction is consistent with hypothesis 5. Comparison and contrast between unrelated species is expected to allow a more robust extrapolation of the observed pattern.

## Materials and Methods

### Species

*Alternanthera philoxeroides* is a serious weed in the United States, China, Australia, New Zealand, India, and many Southeast Asian countries. It was first recorded in Australia in 1946 (National Herbarium of New South Wales record). It has subsequently invaded primarily aquatic to mesophytic terrestrial habitats in central coastal New South Wales [37], including water bodies, banks of waterways, swamps, floodplain pastures and in some dry, elevated locations [38]. Its abundant growth is capable of excluding native vegetation, blocking drainage and irrigation canals [39], reducing water retention [40], and increasing the breeding areas of disease vectors [39], [41]. Reproduction is entirely vegetative in its introduced range [41], [42]. *Phyla canescens* was introduced into Australia by the 1930's and has subsequently become a major weed in riparian habitats, wetlands, and floodplains in the Murray-Darling Basin [32]. It reduces stocking rates, draws moisture from deep in the soil profile, inhibits regeneration of native tree and increases the amount of erosion and soil slumping along river banks [32], [43], [44]. *Phyla canescens* produces seeds prolifically in favourable conditions [45] and propagates vegetatively through stem fragmentation [32].

### Greenhouse experiment

Source material for *Phyla canescens* and *Alternanthera philoxeroides* was collected, respectively, from St Ruth's Reserve near Dalby (27°20′04″S, 151°14′38″E, Queensland, Australia) and the town of Griffith (34°17′21″S, 146°2′37″E, New South Wales, Australia). No specific permits were required for collecting *P. canescens* and *A. philoxeroides* in these locations, which are not privately-owned in any way. Although St. Ruth's reserve is a protected area, permission is required only for collecting native species; the town of Griffith is not a protected area. These field studies did not involve endangered or protected species.

Collected materials (12 individuals of each species) were propagated vegetatively in a greenhouse at the CSIRO Long Pocket Laboratory (Brisbane, Australia). Clonal fragments were planted in seedling pots in November 2006 and subsequently transplanted to 15×15 cm (diameter×height) plastic pots. These plants were allowed to grow roots at multiple nodes and developed into a dense patch within the pot. Plants were trimmed to similar size in April 2007, transplanted to 25×25 cm pots and allowed one month additional growth. Twelve of these plants with similar size (each with at least 30 branches) were then selected for use in the experiment [35]. They represented well-established mother plants consisting of groups of interconnected ramets colonizing a favourable high-light patch. All plants were grown in a 3∶1 blend of commercial potting mixer (H40, including composted pine bark and washed river sand, Centenary Landscape, Brisbane, Australia) and sand. Each litre contained 4 g Osmocote, 1 g Osmoform (Scott, Marysville OH, USA), 0.5 g urea formaldehyde, 0.5 gm IBDU and 1 g (3∶1 respectively) CaSO_4_:MgSO_4_ mix as fertilizer. The amount of total nitrogen (N), phosphoric pentoxide (P_2_O_5_), and potassium oxide (K_2_O) supplied in one litre of medium were 1.10 g, 0.38 g, and 0.55 g, respectively. An additional 8 g Osmocote was supplied to parent plants within two months (during the experiment). This medium and nutrient level is commonly used for fast-growing herbaceous species in the nursery industry and is considered favourable for the growth of both *A. philoxeroides* and *P. canescens* (G. Fichera, personal communication).

**Figure 1:**
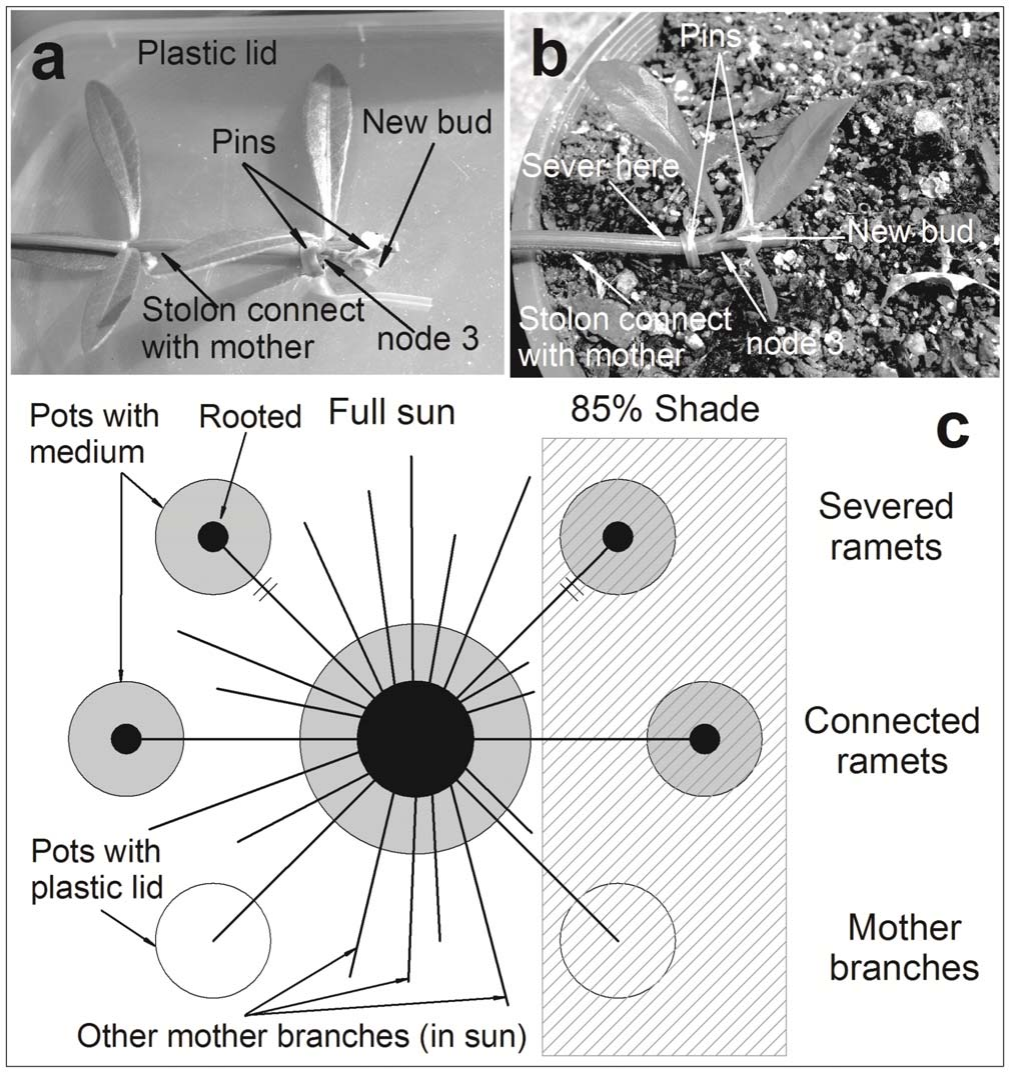
Schematic representation of the experimental design. The set up of unrooted mother branches (a) and connected and severed daughter ramets (b) are shown for *A. philoxeroides*. Each experimental unit consists of one mother plant, two severed daughter ramets, two connected daughter ramets, and two unrooted mother branches (c). The ramets and unrooted branches were subject to either full sun or 85% shade. The mother plant (including all stolons in the pot and unused branches) was exposed to full sun.

To simulate the vegetative expansion of the two weeds from established patches, six well-extended stems were randomly selected for each mother plant. The top two nodes of these stems were removed because inundation treatment that stimulates root growth at the node to facilitate ramet establishment (see below) often damages the apical tissue, especially for *P. canescens*. Two stems were fixed at the third node from their apices on a 15 cm pot that was covered by a plastic lid to avoid root growth (Figure 1a). Shoots that developed from buds based at the third node of these two stems were mother branches that were compared with connected daughter ramets in this experiment. The other four stems were used to establish daughter ramets. The third node of these four branches was placed in a saucer with water to encourage root generation. All nodes had developed several roots (1–2 cm length) after four days. The rooted nodes were potted in a 15 cm plastic pot and fixed in place with two pins (Figure 1b). Ramets developed from new roots and buds based at the third node. Growing conditions were the same as that used to grow the mother plants, except that a 1∶3 blend of commercial potting mixture and sand was used for more effective root harvesting. Compared with the added nutrient, the medium composition contained only trace amounts of nutrients, so difference in the medium composition was not likely to result in differential nutrient availability for mother plants and daughter ramets.

After another six days, all potted nodes rooted effectively (examined by lightly shaking the node) and had sprouted shoots. Severance and light treatments were applied on May 1^st^ 2007, immediately after all potted nodes were confirmed to be successfully rooted. Two daughter ramets were severed from each mother (Figure 1b), so each experimental unit consisted of one mother plant, two connected ramets, two severed ramets, and two branches. Two light treatments were set: full sun and shaded by 85% shade cloth (li-250A, LI-COR, Lincoln, Nebraska, USA) (Figure 1c). One connected ramet, one severed ramet, and one mother branch of the same mother plant were located in each light treatment. The mother plant (including all other branches and stolons) was exposed in full sun. All 12 experimental units of each species were randomly located on benches in a greenhouse and watered to field capacity on a daily basis. Plants were then monitored for eight weeks before harvest (May 1^st^–June 25^th^, 2007). The photosynthetic photon flux density (PPFD) in the greenhouse was 1200–1400 µmol m^−2^ s^−1^ on a sunny day (Hobo UA002-64, Onset Computer Cooperation, Bourne, MA, USA), and the night and day temperature was 10–17°C and 25–32°C, respectively, during the experiment period.

Our design differs from most previous studies in that the ramets and branches exposed to contrasting light treatments were connected to the same mother. Comparing with the more common design that uses one mother supporting one daughter in each of the treatments and controls, our design has the advantage of excluding the variation caused by using different mothers in different treatments and controls. In our experiment, mother plants appeared to have sufficient resources to support two ramets and two branches, as evidenced by their vigorous growth; the average aboveground biomass of mother plants was 10 and 9 times, respectively for *A. philoxeroides* and *P. canescens*, of the sum of connected ramets and branches. Due to this size difference and sufficient resource supply of mother plants, we assumed that studied branches and ramets would not interfere with the physiological and growth status of the mother plant or between each other, and that any difference in morphology and growth between ramets and branches could be attributable to an allocation strategy, rather than the resource limitation, of the mother plant.

### Measurements

All branches and daughter ramets were harvested after eight weeks of growth. Plant biomass was separated into shoots and roots, dried in an oven at 60°C for at least 48 hours to constant weight, and weighed. The root to shoot (R∶S) ratio was calculated by dividing the below ground biomass by above ground biomass. During the harvest, six randomly selected, visually mature (well-expanded, with developed wax surface) leaves of each ramet/branch were collected and scanned to determine leaf size and morphology (defined as length to width ratio) (Photoshop, Adobe, San Jose, CA, USA). Leaf mass per area (LMA) was calculated from leaf area and dry weight. The total length, and the length and diameter of the fourth internode (between the 4^th^ and 5^th^ node) of the main stem (defined as the longest stem) was measured.

To examine the effect of clonal integration on the growth and light acclimation of ramets at different development stages, the total stem number (defined as a branch having at least one node with fully expanded leaves and with the apical bud visible) and ramet height (vertical distance from the surface of medium to the tallest tip of the plant) of all daughter ramets were measured after 8, 26 and 43 days of growth.

### Statistical Analyses

The effect of light and rooting on the traits of stolons connected with the mother plant (branches and connected ramets) and the effect of light and connection on the traits of daughter ramets was analysed through ANOVA. Each analysis was based on a split-plot design; each experimental unit was treated as a “whole plot”; each whole plot was split into two subplots on which different light treatments were added. This design accounted for the effect of the mother plant and the location in the greenhouse before the effects of main factors were assessed. Factorial ANOVA for split plot design was used to test the main effect and the interaction on all traits after the random effect of plant was fitted. The mean square values of connection (or rooting)×plant, light×plant and connection (or rooting)×light×plant were used as error terms to test the effect of connection (or rooting), light and connection (or rooting) by light interaction, respectively. The effect of plant was not assessed in this design because there was no replicate for plant term. Multiple comparisons between treatments were made using Bonferroni tests. Due to the sensitivity of factorial ANOVA for split-plot design to unbalanced sample number, only nine experimental units of *P. canescens* was used in analysis because severed, shaded ramets in the other three units died. A repeated measures ANOVA for split plot design was conducted to assess the temporal changes of stem number and ramet height. Principal component analysis (PCA) was implemented to illustrate the overall reaction norms of connected and severed ramets. All analyses were conducted with Datadesk 6.0 (Data Description Inc., Ithaca, NY, USA). Biomass data were square root transformed to fulfil assumptions of normality and homoscedasticity.

Phenotypic plasticity of ramets and branches were assessed using the index of phenotypic plasticity (the difference between the highest and lowest mean values of all treatments divided by the maximum mean value, 0<PPI<1) for each trait. This allows the plasticity of variables with different units and contrasting variation ranges to be compared [46]. Mean phenotypic plasticity (MPP) of all traits were calculated by averaging PPI of individual traits and was then compared between branches, connected ramets and severed ramets using the non-parametric Wilcoxon signed-rank test [46].

## Results

### The effect of clonal integration on traits and trait light reaction norm

#### Growth and morphological traits

**Figure 2:**
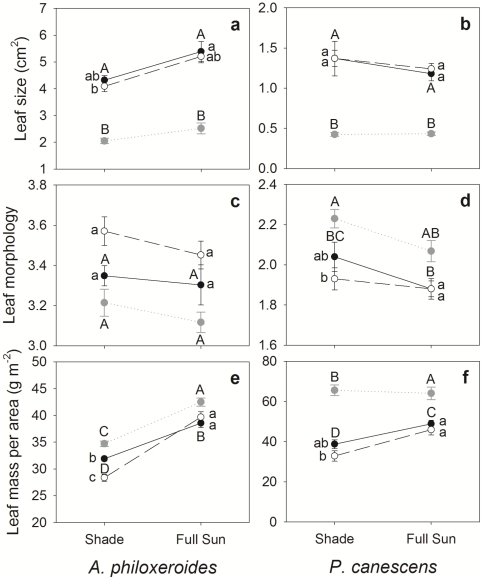
Reaction norms of leaf characters of connected ramets (black filled circle and solid line), severed ramets (open circle and dashed line) and unrooted branches (grey solid circle and dotted line) of *A. philoxeroides* (left panel) and *P. canescens* (right panel). Values are means (±SE), n = 12 or 9. Different letters indicate significant differences (*P*<0.05) between branches and connected ramets (capitals) and between connected and severed ramets (non-capitals) (Bonferroni tests).

**Figure 3:**
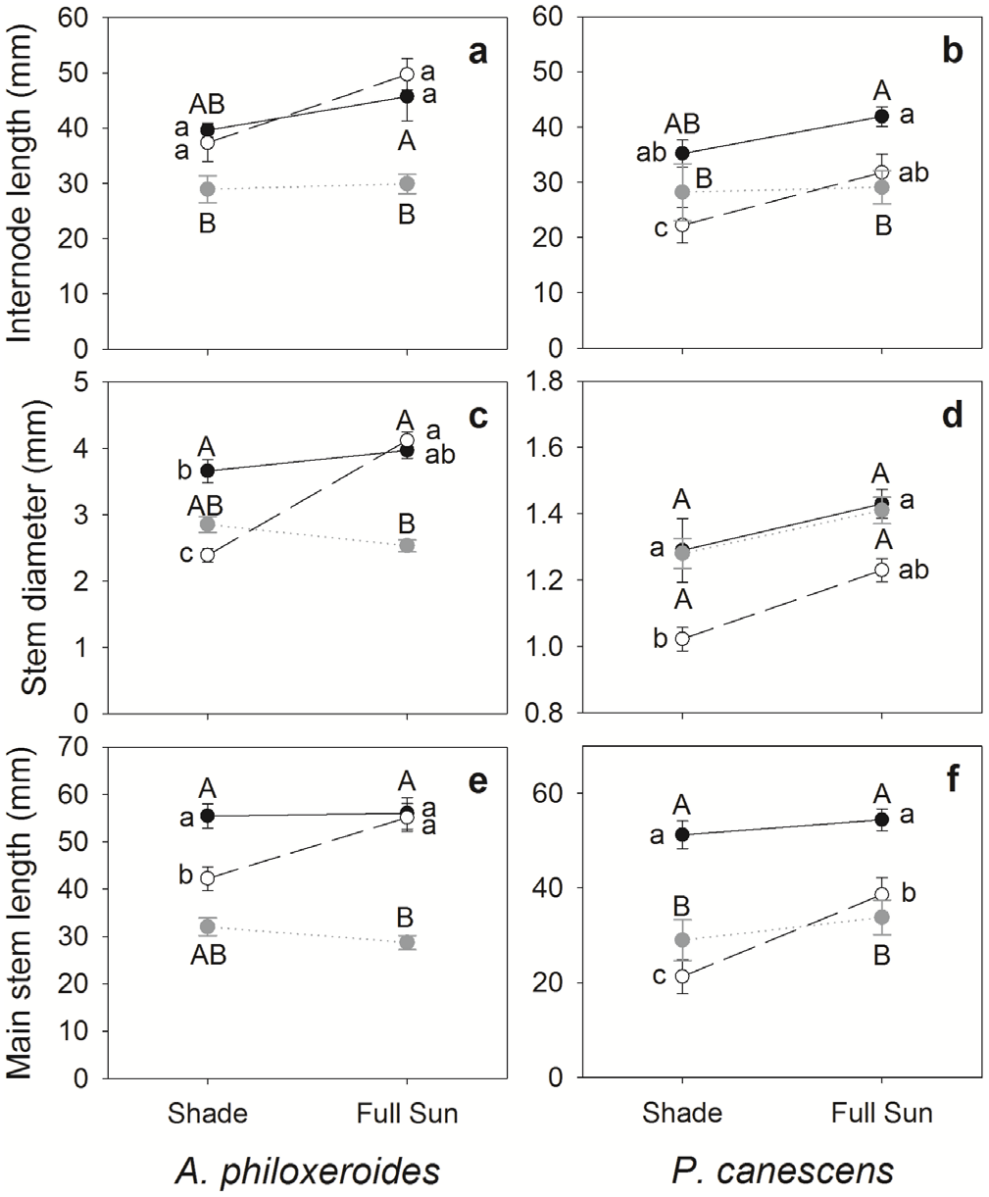
Reaction norms of stem characters of connected ramets (black filled circle and solid line), severed ramets (open circle and dashed line) and unrooted branches (grey solid circle) of *A. philoxeroides* (left panel) and *P. canescens* (right panel). Values are means (±SE), n = 12 or 9. Different letters indicate significant differences (*P*<0.05) between branches and connected ramets (capitals) and between connected and severed ramets (non-capitals) (Bonferroni tests).

**Figure 4:**
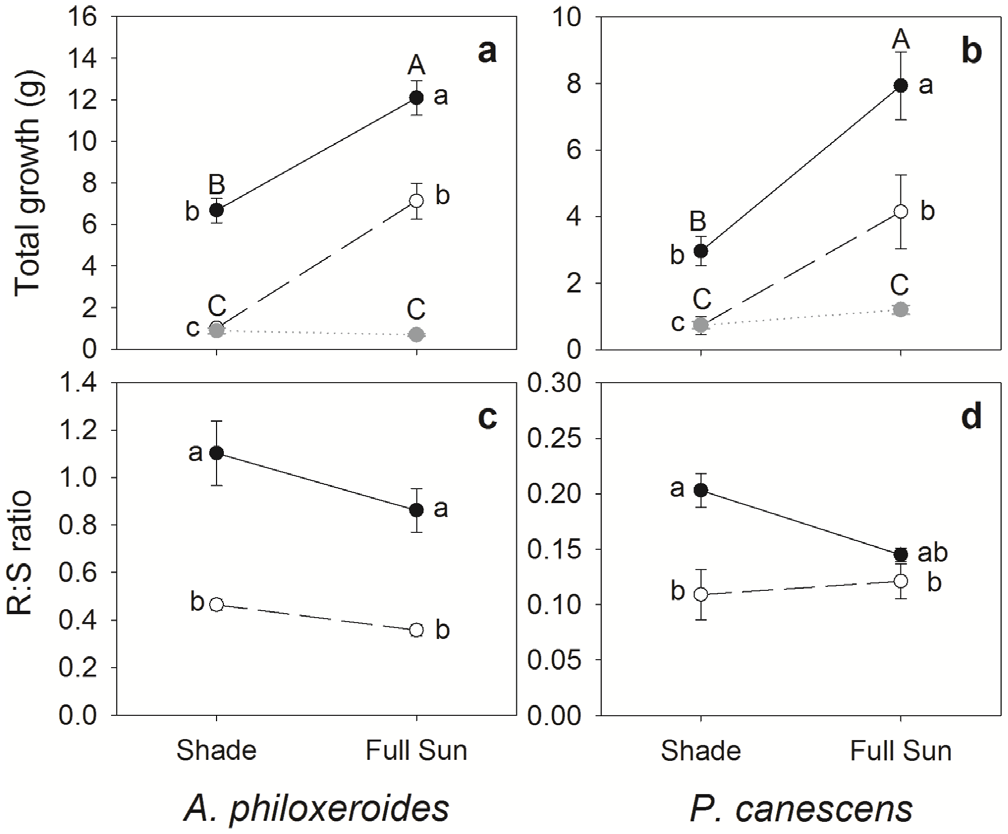
Reaction norms of total biomass and the ramet root to shoot ratios of *A. philoxeroides* (left panel) and *P. canescens* (right panel) connected ramets (black filled circle and solid line), severed ramets (open circle and dashed line) and unrooted branches (grey solid circle and dotted line). Values are means (±SE), n = 12 or 9.

Light effect, as expected, was significant for many traits of both species (7 for *A. philoxeroides* and 4 for *P. canescens*, Table 1). Clonal integration had a significant effect on five traits of *A. philoxeroides*, and four traits for *P. canescens* (Table 1). Compared with severed ramets, connected ramets of *A. philoxeroides* produced wider leaves (reflected by a higher length to width ratio, Figure 2c), whereas connected ramets of *P. canescens* had longer internodes and significantly higher LMA (Figure 2f, 3b). Connected ramets of both species displayed wider and longer stems (Figure 3c–f), greater total biomass, and higher R∶S ratios (Figure 4). Thus, our hypothesis (1) that connected ramets would grow faster than severed ramets is supported.

**Table 1:** ANOVA results of the morphological and growth traits.

Effect	Leaf characters			Stem characters			Growth and allocation	
	Leaf size	Leaf morphology	Leaf mass per area	Internode length	Stem diameter	Main stem length	Total growth	R∶S ratio
*A. philoxeroides*								
Connection	0.47	**0.02**	0.06	0.79	**0.005**	**0.007**	**<0.001**	**<0.001**
Light	**0.002**	0.31	**<0.001**	**0.04**	**<0.001**	**0.03**	**<0.001**	**0.008**
C×L	0.93	0.62	**0.003**	0.37	**<0.001**	**0.04**	**0.002**	0.29
*P. canescens*								
Connection	0.84	0.32	**0.006**	**0.003**	**0.005**	**<0.001**	**<0.0001**	**0.002**
Light	0.34	0.07	**0.001**	**0.008**	0.06	**0.02**	**<0.0001**	0.18
C×L	0.74	0.09	0.50	0.48	0.15	**0.01**	0.46	0.06

Comparisons were made between connected and severed ramets. *P*-values of effects of connection, light, and connection by light interaction are shown. Bold fonts indicate statistically significant effects (*P*<0.05).

As indicated by the significance level of the connection×light interaction, the influence of clonal integration on the light reaction norms of traits was different between the two species. Four traits, i.e. LMA, stem diameter, main stem length, and total growth of the ramets, were significantly influenced by the interaction for *A. philoxeriodes* (Table 1), with severed ramets displaying greater light response than connected ramets (Figures 2, 3, and 4). In particular, sun: shade biomass ratio was 7 for severed ramets and only 1.8 for connected ramets. In contrast, the interaction was not significant for most traits of *P. canescens*, except the main stem length (Table 1, Figure 3). Thus, our hypothesis (2) that clonal integration would more greatly promote the growth of shaded ramets is only supported for *A. philoxeroides*.

#### Temporal pattern

**Figure 5:**
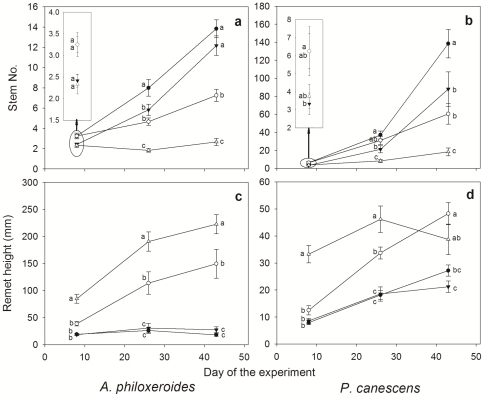
The growth trajectory of stem number and ramet height of connected and severed ramets of *A. philoxeroides* (left panel) and *P. canescens* (right panel) in full sun and 85% shade. The symbols are: filled circle – connected, full sun; open circle – connected, shade; filled triangle – severed, full sun; open triangle – severed, shade. Values are means (±SE), n = 12 or 9. Different letters indicate significant differences (*P*<0.05) during each measurement (Bonferroni tests).

During the first two weeks of the experiment, clonal integration overwhelmed the light effect in shaping the growth of new stems in both species, supporting our hypothesis (3) that clonal integration rather than light effect would dominate the growth of daughter ramets in the early development stage. After eight days connected ramets produced more stems than severed ramets, while the effect of light and the interaction was not significant (Table 2); the connected ramets, even if in shade, grew more stems than severed ramets in full sun (Figure 5a, b). The light effect was significant after 26 and 43 days, although the effect of clonal integration remained significant (Table 2); for both connected and severed ramets, the stem number in the full sun treatment exceeded that in shade (Figure 5a, b). After 43 days clonal integration partially evened out the increase in number of stems under contrasting light conditions for *A. philoxeroides* (indicated by a significant connection×light interaction), but not for *P. canescens* (Table 2, Figure 5a, b). Over the whole experiment, repeated measures ANOVA suggested that the effects of connection, light, time and their two-way interactions were all significant (*P*≤0.05) for *A. philoxeroides*, but the connection×light interaction was insignificant for *P. canescens* (Table 3).

**Table 2:** The effect of clonal integration, light, and time on the stem number and ramet height observed throughout the experiment.

Effect	Stem No.			Ramet height		
	Day 8	Day 26	Day 43^th^	Day 8	Day 26	Day 43
*A. philoxeroides*						
Connection	**<0.001**	**<0.001**	**<0.001**	**<0.001**	**0.03**	**0.03**
Light	0.83	**<0.001**	**<0.001**	**<0.001**	**<0.001**	**<0.001**
C×L	0.87	0.43	**0.03**	**<0.001**	**0.03**	0.06
*P. canescens*						
Connection	**0.01**	**<0.001**	**0.002**	**<0.001**	**0.04**	**0.05**
Light	0.61	**0.04**	**<0.001**	**<0.001**	**<0.001**	**<0.001**
C×L	0.94	0.17	0.56	**<0.001**	**0.01**	0.64

ANOVA results are shown respectively for three measurements made after 8, 26 and 43 days. *P*-values of effects of connection, light and connection by light interactions are given. Bold fonts indicate statistically significant effects (*P*<0.05).

**Table 3:** The effect of clonal integration, light, and time on the stem number and ramet height observed throughout the experiment.

Effect	Stem No.		Ramet height	
	*A. philoxeroides*	*P. canescens*	*A. philoxeroides*	*P. canescens*
Connection	**<0.001**	**<0.001**	**0.005**	0.10
Light	**<0.001**	**0.002**	**<0.001**	**<0.001**
C×L	**0.05**	0.93	**0.009**	**0.009**
Time	**<0.001**	**<0.001**	**<0.001**	**<0.001**
C×T	**0.006**	**<0.001**	0.44	**<0.001**
L×T	**<0.001**	**<0.001**	**<0.001**	0.38
C×L×T	0.06	0.41	0.63	**0.01**

Repeated measures ANOVA results are shown. *P*-values of effects of connection, light, time and interactions are given. Bold fonts indicate statistically significant effects (*P*<0.05).

The ramet height of *A. philoxeroides* and *P. canescens* showed the same light reaction norm. In the early stage of establishment (Day 8), there was a very significant connection×light interaction (Table 2). That is, severed ramets grew 2–3 times taller in shade than in full sun while contrasting light did not alter the height of connected ramets (Figure 5c, d). Overall, the significance level of the connection effect and the connection×light interaction decreased throughout the experiment (Table 2). By day 43 approximately 20% of the severed, shaded ramets laid flat, possibly because the vascular supporting tissue could no longer support vertical growth. This mechanical limit contributed to the decrease of ramet height in the third measurement and the lack of connection effect in repeated measures ANOVA for *P. canescens* (Table 3).

#### PCA and plasticity

**Figure 6:**
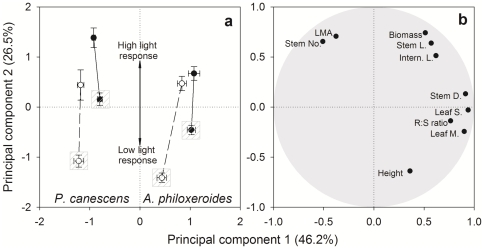
Principal component analysis based on traits. Principal components diagram of treatments (a) show the overall light reaction norm of morphological and growth traits. Values are means (±SE), n = 12 or 9. The first principal component separate *A. philoxeroides* (left) and *P. canescens* (right), and connected (filled circle and solid line) and severed (open circle and dashed line) ramets. The second principal component reflects the light response of ramets, with shade treatments marked by gray dashed squares. Composition of the first two principal components (loading values of traits) is shown (b).

Principal component analysis was conducted using ten traits (Figure 6). The first two principal components (PC) explained 75% of ramet trait variation in the two species. PC1 is mainly associated with leaf size and morphology, stem diameter, and R∶S ratio, separating the two species (by leaf size and morphology) and connected *vs.* severed ramets (by stem diameter and R∶S ratio) (Figure 6). The overall light reaction norm of morphological and growth traits is defined by PC2 (Figure 6a), which is related to total biomass, LMA, stem number, ramet height, main stem length, and internode length (Figure 6b). In general, connected ramets showed better growth (greater biomass and wider stems), higher allocation to root biomass (R∶S), and a more creeping and elongation habit (lower height, longer internode and main stem). Overall, the light reaction norm of connected vs. severed ramets exhibited a similar magnitude in *P. canescens*, whereas for *A. philoxeroides* the reaction norm of severed ramets had much greater magnitude than that of connected ramets. In particular, the difference between connected and disconnected ramets in full sun treatments was less pronounced for *A. philoxeroides* than for *P. canescens*.

Phenotypic plasticity in response to light was compared between connected ramets and severed ramets using ten traits (Table 4). Leaf morphology generally exhibited little plasticity. Connected and severed ramets produced similar PPI for leaf size, leaf morphology, R∶S and maximum height in *A. philoxeroides*, but not in *P. canescens*. The other six traits showed a consistent effect of connection in both species, i.e. severed ramets had higher plasticity than connected ones. Overall, the influence of clonal integration on plasticity was larger in *A. philoxeroides* than in *P. canescens*. Connected ramets of *A. philoxeroides* displayed higher MPP (10-traits) than severed ramets, but the connection effect on the MPP was not significant for *P. canescens*. This result agrees with the PCA analysis, and these two analyses support our hypothesis (4) that connected ramets would display lower plasticity to the light treatment than severed ramets, although it was only evident for *A. philoxeroides.*

**Table 4:** Phenotypic plasticity index (PPI) measured for non-rooted branches, and connected and severed ramets.

Traits		*A. philoxeroides*			*P. canescens*		
		Branch	Connected	Severed	Branch	Connected	Severed
Leaf size		0.19	0.20	0.21	0.04	0.14	0.09
Leaf morphology		0.03	0.01	0.03	0.07	0.08	0.03
LMA		0.18	0.17	0.29	0.22	0.21	0.28
Internode length		0.02	0.13	0.25	0.06	0.16	0.30
Stem diameter		0.12	0.08	0.42	0.08	0.02	0.17
Main stem length		0.11	0.01	0.23	0.18	0.06	0.45
Total biomass		0.22	0.45	0.86	0.43	0.63	0.83
R∶S		–	0.22	0.23	–	0.29	0.09
Stem No.		–	0.48	0.78	–	0.56	0.79
Maximum height		–	0.82	0.82	–	0.43	0.57
MPP	(7-trait)	0.12^a^	0.15^a^	0.33^b^	0.15^a^	0.18^a^	0.31^b^
	(10-trait)	–	0.26^a^	0.41^b^	–	0.26^a^	0.36^a^

The mean phenotypic plasticity (MPP) of all traits was calculated and compared among the branches and the ramets. MPP values accompanied by different letters are significantly different at *P* = 0.05 level when compared in pairs (Wilcoxon signed-rank test).

### Trait differences between unrooted branches and connected ramets

When comparing traits between branches and connected ramets, *A. philoxeroides* and *P. canescens* showed a consistent pattern. In both species, branches displayed different morphological and growth traits from connected ramets (reflected by a significant rooting effect for most traits, Table 5). Compared with connected ramets, branches generally had smaller and thicker (indicated by higher LMA) leaves (Figure 2a, b, e, f) and shorter internodes and main stems (Figure 3a, b, e, f). Light reaction norms of most leaf and stem characters were similar between branches and connected ramets (Figures 2, 3, and 4). In both species, branches showed much less growth than connected ramets, regardless of light level, and there was a very significant effect of a rooting×light interaction on total growth. This result supports our hypothesis (5) that connected ramets would display greater growth than mother branches. Branches and connected ramets of both species also displayed similar patterns in plasticity to contrasting light (Table 4, 7-trait MPP). Branches exhibited lower plasticity than connected ramets for internode length and total biomass, but higher values for stem diameter and main stem length; the differences in leaf characters were negligible.

**Table 5:** ANOVA results of the morphological and growth traits.

Effect	Leaf characters			Stem characters			Total growth
	Leaf size	Leaf morphology	Leaf mass per area	Internode length	Stem diameter	Main stem length	
*A. philoxeroides*							
Rooting	**0.003**	**0.01**	**<0.001**	**<0.001**	**<0.001**	**<0.001**	**<0.001**
Light	**0.004**	0.28	**<0.001**	0.32	0.99	0.53	**<0.001**
R×L	0.28	0.72	0.31	0.36	**0.02**	0.35	**<0.001**
*P. canescens*							
Rooting	**<0.001**	**0.05**	**<0.001**	**0.03**	0.21	**<0.001**	**<0.001**
Light	0.23	**0.003**	**<0.001**	0.12	0.07	0.23	**<0.001**
R×L	0.15	0.96	**0.003**	0.20	0.34	0.52	**0.001**

Comparisons were made between non-rooted branches and connected ramets. *P*-values of effects of rooting, light, and rooting by light interaction are shown. Bold fonts indicate statistically significant effects (*P*<0.05).

## Discussion

Clonal integration affected morphological and growth responses to contrasting light conditions differently in *A. philoxeroides* and *P. canescens*. For *A. philoxeroides*, clonal integration evened out the morphological plasticity to light conditions and greatly increased the growth of shaded ramets compared to ramets grown in full sun. These connection×light interactions were largely absent for *P. canescens*. Despite this species-specific effect, clonal integration had a strong, promoting effect on ramet growth in both high and low light conditions. The contribution of clonal integration during their early development stage, especially on ramet growth, overwhelmed the light effect and may therefore facilitate ramet establishment in high competition habitats. Finally, connected ramets grew much faster than mother branches in both high and low light treatments, despite similar morphological response patterns to contrasting light levels. This suggests that mother plants prioritise daughter ramets over their own branches when allocating resources.

### Species-specific clonal integration effect on *A. philoxeroides* and *P. canescens*

Clonal integration generally improves the growth of ramets but the advantages vary between species [20], [23]. In a previous study, we found species-specific patterns in maternal subsidy and the effect of clonal integration on growth in these two species [35]. For *A. philoxeroides*, shaded daughter ramets received more maternal subsidy than full sun ramets, and their growth was greater. For *P. canescens*, maternal subsidy to daughters grown in contrasting light conditions was not significantly different and clonal integration therefore affected growth of shaded and unshaded ramets similarly. In this study, we confirmed this conclusion. In addition, we found that clonal integration generated a species-specific effect on the light response of morphological traits and phenotypic plasticity. In addition to growth in total biomass and the number of stems, clonal integration also evened out the shade response of LMA and stem diameter of *A. philoxeroides*, but not for *P. canescens*. This covariance suggests that the light response of biomass growth is, at least partially, mediated by the response of morphological traits. Although model simulation suggests that translocation of resources over the spacer system could alter clonal morphology [15], the physiological mechanism of how the light response pattern of these morphological traits is affected by clonal integration is not clear. Further studies are needed to address whether the light response of these morphological traits is directly or indirectly affected by the species-specific pattern of maternal subsidy.

Overall, the species-specific effect of clonal integration on ramet growth, maternal subsidy and morphological traits indicates that *A. philoxeroides* takes a more proactive approach in terms of establishing daughter ramets in unfavourable, low- light habitats. For *A. philoxeroides*, a species with strong underground vegetative propagation capacity [47], [48], this strategy may be advantageous. Once established the thickened roots of *A. philoxeroides* provide a major resource pool that buffers the species against unfavourable conditions and generates new ramets when conditions become favourable. In this case, even if above-ground material and maternal connections are destroyed, established roots of *A. philoxeroides* ramets constitute a “propagule bank” which is able to rapidly recolonise when conditions become suitable. In contrast, roots of *P. canescens* can't generate ramets [49] so establishing ramets under low-light conditions may not be advantageous. This is supported by the unbiased clonal integration effect on ramets under different light conditions that we observed. This hypothesis needs to be tested in future studies.

### Morphological and growth responses to light

Clonal plants can display highly plastic changes in ramet morphology and this is believed to enable effective exploitation of local concentrations of essential resources [50]. Significant morphological plasticity to contrasting light condition was observed in both *A.philoxeroides* and *P. canescens*. Both species displayed some typical characteristics of light acclimation, such as thicker leaves associated with increased photosynthetic capacity in full sun [35] and vertically elongated ramet stems that may lift leaf blades to a higher light zone. It is interesting that the light response of some traits is inconsistent with some generally observed patterns in other clonal plants. For example, shaded leaves usually have larger lamina with lower LMA to promote effective light exploitation and this acclimation has been observed in clonal plants [51], [52]. However, in our study, the response of leaf size is not significant for *P. canescens* while *A. philoxeroides* displayed expanded leaf size in full sun. Similarly, the light response pattern of internode length is opposite to what has been consistently observed in previous studies. In general, clonal plants display shortened internodes under high photon flux densities (see review [50]), which allows more branches and leaves to be located in favourable habitat patches. However, we observed that internode length of *A. philoxeroides* and *P. canescens* increased in full sun. These characteristics (expanded leaf and elongated internode in full sun) may be associated with the rapid colonisation ability of these two weeds in habitats with high light availability and low competition levels, and thus contribute to their competitive advantage over other species. Comparing leaf and stem light response patterns of *A. philoxeroides* and *P. canescens* with some clonal native species and clonal, non-invasive exotic species would help test whether this is an important trait for invasiveness.

A plastic response in the lengths of stolon internodes and petioles is hypothesised to be important for the capture of light in a patchy environment [53]. Spacer extension may enable the plant to escape from shaded patches [54], while petiole elongation may lift shaded leaves into a higher light zone [51]. For *A. philoxeroides* and *P. canescens*, connected and severed ramets used these two different shade acclimation strategies, respectively, to escape shaded conditions. In the shade treatment, connected ramets developed longer main stems than severed ramets, whereas the stems of severed ramets displayed greater vertical elongation. This phenomenon indicates that severed ramets tend to acclimate to shade by attempting to lift shaded leaves into higher light zones, while connected ramets tend to “escape” the shaded zone by faster horizontal expansion of its spacers. Maternal subsidies to connected ramets may facilitate increased stem growth rates and facilitate the “escape” strategy. These contrasting morphological acclimation patterns to shade in connected and severed ramets, although being supported by some model simulation [54], have not been reported in previous empirical studies.

The root-shoot (R∶S) ratio of connected ramets observed in this study supports the optimal biomass allocation theory and the division of labour theory for clonal plants. Non-clonal plants usually allocate biomass towards acquiring resources that are limiting [55]. In contrast, clonal plants often display divisions of labour in resource-acquisition duties, with ramets specialized in capturing locally abundant resources [14], [56]. When grown in shade, connected ramets of both species allocated more biomass to the roots (although statistically insignificant for *P. canescens*), indicating labour division among ramets under different resource conditions. The higher R∶S ratio of connected ramets may enhance the acquisition of soil resources to balance the photosynthate subsidy from mother plants.

### Clonal integration benefits ramet growth during early development

Previous studies consistently show that clonal integration promotes the establishment of ramets, especially in unfavourable habitats [19], [20], [23], [25], [29], [57], [58], and our study also supports this. However, such studies rarely consider the effect of time on clonal integration. Our study found that the effect of clonal integration in both studied species was most important in the earliest stage of ramet establishment. Enhancement of growth due to clonal integration was observed earlier than the light effect (8 *vs*. 26 days after ramets rooted), indicating that maternal subsidy was particularly beneficial to the growth of connected daughter ramets during their early development stage. Furthermore, vertical stem elongation, a typical plant growth response in light limited environments, was strongly affected by clonal integration after 8 days, whereas the effects of connection and connection×light interactions disappeared after 43 days. These results are consistent with the pattern observed in the ephemeral clonal plant *Calathea marantifolia*, in which the contribution of clonal integration to survival rate and life expectancy of daughter ramets decreased with time [28]. The decreasing influence of clonal integration over time observed in our study may be due to diminished maternal subsidy, increasing assimilation of the daughter ramet, or both. Further study, e.g. whether maternal subsidy would last longer time in stressful environments, is needed to identify underlying mechanisms.

### Branch vs. connected ramets

Few previous studies have compared connected ramets and branches of the mother plant. In this study, we found that branches of *A. philoxeroides* and *P. canescens* displayed were generally smaller (smaller leaves, thinner stems, shorter internodes and main stems) but still displayed similar light reaction norms to connected ramets and. The absence of roots means that branches are not ready for vegetative reproduction or dispersion in terrestrial habitats. However, the shorter internode length of branches probably allows the mother plant to locate more leaves to focus on resource exploitation, inferring a labour division between mother branches and connected ramets. Mother branches also grew more slowly than connected ramets, which suggests that mother plants allocate more resources per module to support the growth of daughter ramets compared to their own branches. Alternatively, underground resources uptaken by roots may promoted the growth of daughter ramets, while this is not possible for the branches. Another interesting observation is that the growth of branches was not affected by light availability and the growth of branches was similar, or even lower than the severed, shaded ramets. This suggests that mother branches are a net “source” that translocate a high proportion of assimilates to storage or tissue growth elsewhere. Branch growth is therefore probably determined at the whole plant resource level and not by the light availability of their local environment.

### Conclusion

In this study, we observed a species-specific effect of clonal integration on the light reaction norm of morphological and growth traits in *A. philoxeroides* and *P. canescens*. This is in line with the maternal subsidy pattern of these two species displayed in a previous study [35]. Clonal integration overwhelmed light effect on early ramet growth thereby improving the establishment of juvenile ramets of both species in light-limiting environments. Finally, mother plants tend to allocate more resources per module to support the growth of daughter ramets, rather than their own branches. This, together with the higher growth rate of ramets, may contribute to the ability of *A. philoxeroides* and *P. canescens* to successfully colonize native plant communities. In future studies, comparing and contrasting *A. philoxeroides* and *P. canescens* with clonal native species and clonal non-invasive exotic species will further elucidate the importance of clonal integration in the invasiveness of these two plant invaders.
